# Ultrasound–Ethanol Pretreatment-Assisted Enzymatic Method: A Potential Method to Improve the Quality and Yield of *Perilla* Seed Oil

**DOI:** 10.3390/molecules31101608

**Published:** 2026-05-11

**Authors:** Jinhua Shao, Yichun Zhu, Miaomiao Chang, Shengmei Tang, Weizhen Huang, Liyan Jiang

**Affiliations:** College of Chemistry and Bioengineering, Hunan University of Science and Engineering, Yongzhou 425199, China; jinhua0212@163.com (J.S.);

**Keywords:** ultrasound, pretreatment, aqueous enzymatic extraction, *perilla* seed oil, quality analysis

## Abstract

This study investigates the extraction of perilla seed oil using an ultrasound–ethanol pretreatment combined with aqueous enzymatic extraction (UEAEE). By comparing different pretreatment groups, it was found that the combination of ultrasound and ethanol pretreatment yielded the highest oil extraction efficiency from perilla seeds. Meanwhile, scanning electron microscopy (SEM) analysis further revealed pronounced structural disruption in seeds subjected to ultrasound–ethanol pretreatment, including extensive cellular collapse and the formation of numerous irregular pores and fissures, which facilitated subsequent oil release. The parameters of aqueous enzymatic extraction (AEE) were optimized using a Box–Behnken design. The optimal conditions were determined to be a hydrolysis time of 5 h, a reaction temperature of 52 °C, an enzyme concentration of 5%, and a liquid-to-material ratio of 9.5:1 (mL/g). Compared with oils obtained by pressing extraction (PE) and Soxhlet extraction (SE), UEAEE-derived oil exhibited a higher proportion of unsaturated fatty acids, along with lower peroxide and Acid value (AV), indicating superior quality and a reduced susceptibility to rancidity. Moreover, UEAEE oil retained markedly higher levels of micronutrients (carotenoids: 1.23 mg/kg; total flavonoids: 1.04 mg/g; total phenols: 2.79 mg/g) and demonstrated stronger antioxidant activity (DPPH: 80.60%). Overall, these results demonstrate that UEAEE is an efficient and environmentally friendly extraction method that supports the high-value utilization of *perilla* seed oil and aligns with modern green processing principles.

## 1. Introduction

*Perilla frutescens* (L.) Britt., commonly known as *perilla* or red *perilla*, is an annual herb belonging to the genus *Perilla* in the Lamiaceae family [1]. It is widely cultivated in China, Japan, South Korea, and other regions of Asia [2]. *Perilla* seeds have a high oil content (35–45%), and the extracted oil exhibits desirable sensory properties, including a stable color, clear appearance, and a characteristic aroma [3,4]. Notably, *perilla* seed oil is exceptionally rich in polyunsaturated fatty acids, which account for 70.7–83.4% of the total fatty acid content, with α-linolenic acid (ALA) comprising 58.8–70.9% [5]. In addition, it contains a variety of bioactive compounds, such as vitamin E, carotenoids, and phytosterols, which contribute to its potential roles in preventing cardiovascular and neurological diseases, modulating immune function, and exerting anti-inflammatory and anticancer effects [6,7,8]. Consequently, the further development and utilization of perilla seed oil hold considerable promise for applications in the food, pharmaceutical, and health-related industries.

Numerous studies have reported the use of combined extraction techniques and pretreatment methods to enhance the yield and quality of *perilla* seed oil, including microwave-assisted and ultrasound-assisted extraction. Feng et al. [9] employed microwave-assisted n-hexane extraction for *perilla* seed oil, while Li et al. [10] utilized ultrasonic-assisted solvent extraction. In addition, Li et al. [11] optimized ultrasonic-assisted aqueous enzymatic extraction (AEE) for *perilla* seed oil. In recent years, AEE has been recognized as a safe and environmentally sustainable technique that preserves bioactive components while maintaining a high oil yield [12], making it highly promising for further application. At present, ultrasound is most commonly applied during the enzymatic hydrolysis stage of AEE, as its demulsification effect accelerates emulsion breakdown, reduces settling time, and decreases the need for demulsifiers [13]. Ultrasonic treatment also promotes the collision and coalescence of microdroplets, thereby facilitating gravitational oil-water separation and improving extraction efficiency [14]. However, ultrasound may also intensify emulsification in aqueous–oil systems, and the balance between emulsification and demulsification is strongly influenced by factors such as sonication time and temperature [15]. Therefore, applying ultrasonic treatment exclusively during the pretreatment stage may represent a viable strategy, allowing its advantages to be fully utilized while minimizing the risk of exacerbating emulsification.

Ethanol, a renewable and environmentally friendly solvent widely used in oil extraction, can further enhance the process; when combined with ultrasound, it improves oil dissolution efficiency while supporting a greener and more economical extraction pathway [16]. Zhang et al. [15] applied ultrasound–ethanol pretreatment combined with AEE (UEAEE) to hemp seed oil. They found that the pretreatment altered the surface morphology and emulsification state of hemp seeds, rendering the substrate more susceptible to enzymatic attack and promoting the formation of a less stable emulsion, which ultimately enhanced oil yield. AEE is often limited by challenges such as the formation of stable emulsions, high enzyme costs, and difficulties in enzyme recovery and reuse [17]. The combination of ultrasound and ethanol in the pretreatment stage addresses these limitations through a dual mechanism. The physical effects generated by ultrasound mechanically disrupt plant cell wall integrity and increase tissue porosity. This “pre-disruption” effect significantly reduces physical barriers to enzymatic action, enabling more rapid and efficient enzyme–substrate contact and thereby improving conversion efficiency at lower enzyme loadings [18]. Meanwhile, ethanol modulates system polarity and induces protein denaturation at the emulsion interface, thereby destabilizing emulsions at a fundamental level [19]. This synergistic strategy simultaneously enhances enzymatic accessibility through physical disruption and facilitates phase separation through chemical demulsification, ultimately reducing production costs and increasing free oil recovery. Moreover, compared with existing hybrid extraction methods, this approach offers distinct advantages. For instance, ultrasound–microwave synergistic extraction can rapidly increase yield through microwave-induced thermal effects; however, the high temperatures generated may promote lipid oxidation. In addition, such methods often rely on organic solvents such as petroleum ether, raising concerns regarding solvent residues and additional separation costs [20]. In contrast, UEAEE eliminates the need for microwave heating and uses water as the subsequent extraction medium, thereby avoiding the risk of organic solvent residues. This makes it more suitable for the production of high-quality, clean-label oil products. Therefore, ultrasound–ethanol pretreatment holds significant research value and application potential.

Existing pretreatment methods for *perilla* seeds primarily include microwave treatment [21], infrared baking [22], and freeze–thaw pretreatment [23]. However, to date, no studies have reported the use of an ultrasound–ethanol coupled pretreatment strategy. Therefore, this work introduces an ultrasound–ethanol pretreatment to assist AEE of *perilla* seed oil. Based on the optimization of extraction parameters, the study further compares the quality of the obtained oil with that produced by pressing extraction (PE) and Soxhlet extraction (SE). The aim is to provide a technological foundation for the industrial application and high-value utilization of *perilla* seed oil.

## 2. Results and Analysis

### 2.1. Effect of Ultrasound–Ethanol Pretreatment on Oil Yield of Perilla Seeds

#### 2.1.1. Effects of Different Pretreatment Methods Assisted with AEE on Oil Yield of *Perilla* Seeds

**Figure 1:**
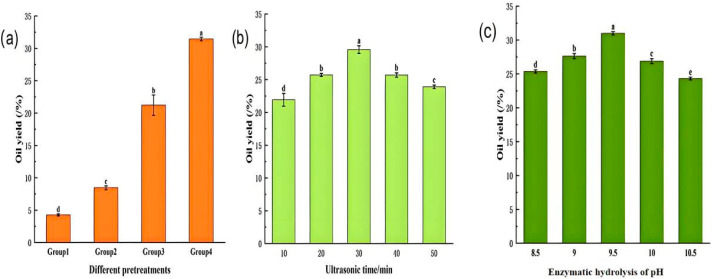
The effects of different pretreatment methods on the oil yield of *perilla* seed oil (**a**), effects of ultrasonication time on the oil yield of *perilla* seed oil (**b**), effects of enzymatic hydrolysis pH on the oil yield of *perilla* seed oil (**c**) (under the conditions of ultrasonic power of 200 W, ultrasonic time of 30 min, and 60% ethanol). Note: (1). Experimental Group 1: Extracted oil without ethanol addition and without ultrasonic pretreatment; Experimental Group 2: Extracted oil with ethanol addition but without ultrasonic pretreatment; Experimental Group 3: Extracted oil without ethanol addition but with ultrasonic pretreatment; Experimental Group 4: Extracted oil with both ethanol addition and ultrasonic pretreatment. (2). Different lowercase letters in the Figure indicate statistically significant differences (*p* < 0.05). The same notations apply to the following all Figures.

Figure 1a illustrates the effects of different pretreatment methods on the oil yield of *perilla* seeds. The oil yield followed the order: Experimental Group I (no ethanol, no ultrasonic pretreatment) < Group II (ethanol addition only) < Group III (ultrasonic pretreatment only) < Group IV (combined ethanol addition and ultrasonic pretreatment). The group without any pretreatment exhibited the lowest oil yield, whereas ultrasonic pretreatment alone increased the oil yield by 16.95%. This improvement is likely attributed to the cavitation effect generated by ultrasound, where cavitation bubbles form and violently collapse during expansion–compression cycles, producing localized shock waves that enhance mass transfer between the solvent and the seed matrix [24]. The cavitation-induced disruption and softening of cell walls facilitate structural degradation, increase solvent permeability, and thereby promote more efficient oil release [25].

Notably, ethanol pretreatment alone also increased the oil yield of *perilla* seeds (Figure 1a), although the effect was more pronounced when combined with ultrasonic pretreatment. Specifically, the oil yield reached 31.44% following ultrasound–ethanol pretreatment. This enhancement can be attributed to ethanol’s ability to penetrate cell walls and dissolve hydrophobic compounds. As ethanol diffuses into the seed matrix, it expands the effective interaction volume; however, spatial constraints within the cellular structure limit the dissolution and transport of amphiphilic substances [15]. Ultrasonic pretreatment generates intense shear forces and turbulence that disrupt plant cell walls, thereby enhancing mass transfer, solvent penetration, and the interaction between the raw material and extraction solvent. These effects collectively facilitate the dissolution and extraction of hydrophobic compounds by ethanol [26,27]. Consequently, UEAEE demonstrates clear advantages for the efficient extraction of *perilla* seed oil.

While ultrasonic pretreatment enhances solvent permeability and facilitates oil release [25], excessively prolonged ultrasonic exposure may reduce enzyme activity and extraction efficiency, and the continuous generation of heat may also degrade oil quality [28]. As shown in Figure 1b, the oil yield of *perilla* seeds initially increased with ultrasonic time, reaching a maximum at 30 min, and then declined with further treatment. At an ultrasonic time of 30 min, the oil yield increased significantly, primarily due to mechanical effects such as cavitation bubble oscillation. These effects generate localized high temperature, high pressure, and strong shear forces, which disrupt cell walls and tissue structures, enhance heat and mass transfer, promote the release of intracellular components, and thereby increase oil yield [29]. However, optimal extraction time is required to achieve maximum oil recovery while maintaining oil quality, as excessive extraction may lead to oil dissolution loss and degradation of seed components [30]. Nevertheless, Li et al. reported that although the extraction yield continued to increase with ultrasonic duration beyond 30 min, the rate of increase gradually diminished and eventually tended toward a plateau [10].

#### 2.1.2. SEM of *Perilla* Seeds After Different Pretreatments

**Figure 2:**
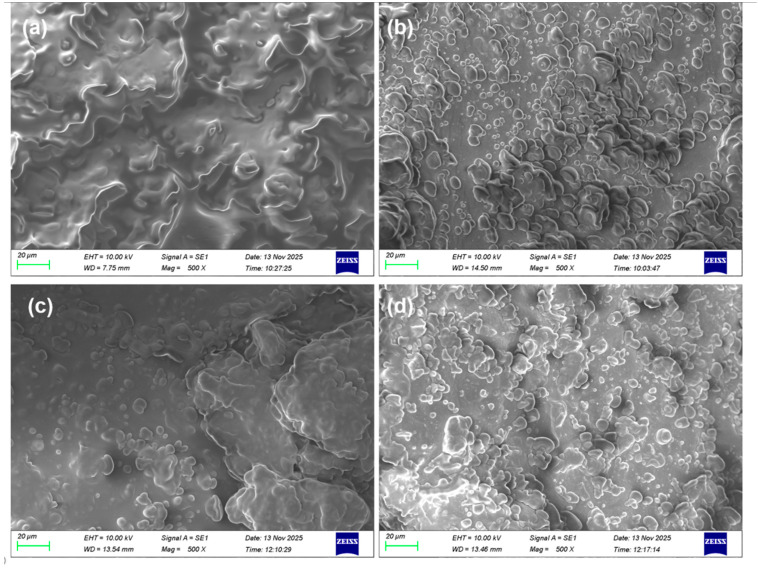
SEM of *perilla* seeds after different pretreatments: no ultrasound and ethanol pretreatment (**a**), ethanol addition without ultrasonic pretreatment (**b**), ultrasonic pretreatment without ethanol (**c**), and ultrasound–ethanol pretreatment (**d**).

Figure 2 presents SEM images depicting the structural changes in *perilla* seeds under different pretreatment conditions: (a) no ultrasound or ethanol pretreatment, (b) ethanol addition without ultrasonic pretreatment, (c) ultrasonic pretreatment without ethanol, and (d) ultrasound–ethanol pretreatment. In Figure 2a, the untreated *perilla* seed powder exhibits intact cellular structures with clearly defined cell boundaries. In Figure 2b, with ethanol addition alone, the overall cellular morphology remains largely preserved, although slight surface shrinkage is observed. In contrast, Figure 2c shows that ultrasonic pretreatment without ethanol induces localized rupture of cell walls, formation of micropores, and surface roughening. The most pronounced structural alterations are observed in Figure 2d, where ultrasound–ethanol pretreatment results in severe collapse of the cellular structure, accompanied by numerous irregular pores and fissures on the surface. These findings are consistent with the SEM observations reported by Wang et al. [31] for *Carya illinoinensis*, where untreated cells remained intact while ultrasonicated samples exhibited irregular pore formation. Overall, these visual evidences underscore the strong disruptive effect of ultrasound–ethanol pretreatment on oilseed cellular architecture and provide direct morphological support for the enhanced extraction efficiency observed in this study.

### 2.2. Analysis of Single-Factor Experiment Results

**Figure 3:**
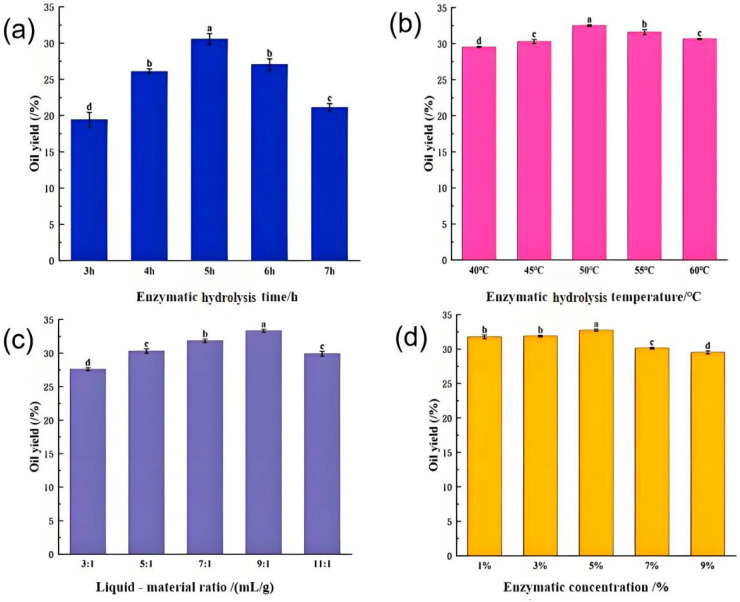
The effect of reaction conditions on the oil yield of *perilla* seed oil. (**a**) enzymatic hydrolysis time, (**b**) enzymatic hydrolysis temperature, (**c**) liquid-to-material ratio, and (**d**) enzyme concentration. (under the conditions of ultrasonic power of 200 W, ultrasonic time of 30 min, and 60% ethanol).

Based on preliminary experiments, single-factor experiments were conducted to optimize the AEE process. The ultrasound-assisted pretreatment conditions were fixed at a power of 200 W, a temperature of 55 °C, and a duration of 30 min, while the ethanol pretreatment concentration was set at 60%. The results are as follows:

Enzymatic hydrolysis time is a critical factor influencing oil release. Insufficient hydrolysis limits the interaction between enzymes and cell wall components, resulting in incomplete oil extraction. Conversely, excessive hydrolysis may degrade product quality and reduce process efficiency; therefore, determining the optimal hydrolysis time is essential. Figure 3a illustrates the effect of enzymatic hydrolysis time on the oil yield of *perilla* seeds. The oil yield steadily increased from 3 to 5 h, reaching a maximum of 30.25% at 5 h. This increase can be attributed to prolonged enzymatic action, which progressively disrupts seed cell structures, thereby facilitating more complete enzyme–substrate interactions and enhanced oil release [32]. Beyond 5 h, the oil yield decreased, likely due to disruption of material balance, substrate depletion, and enzyme inhibition caused by interactions between enzymes and accumulated hydrolysis products [33].

Enzymes generally exhibit optimal activity within specific temperature ranges; in particular, alcalase shows high activity between 45 °C and 60 °C. Figure 3b illustrates the effect of enzymatic hydrolysis temperature on the oil yield of *perilla* seeds. Within the range of 40 °C to 50 °C, the oil yield increased steadily with rising temperature. This trend can be attributed to reduced oil viscosity, increased cell membrane permeability, and enhanced diffusion coefficients at elevated temperatures, all of which promote mass transfer and oil diffusion [17]. The maximum oil yield of 32.44% was achieved at 50 °C, corresponding to the optimal activity of alcalase. Above 50 °C, enzyme activity declined, and the cavitation effect of ultrasound was weakened, leading to reduced mass transfer efficiency and energy utilization [34]. These factors collectively contributed to a decrease in extraction efficiency at higher temperatures.

Figure 3c shows the effect of the liquid-to-material ratio on oil yield. As the liquid-to-material ratio increased, the oil yield initially rose. At low ratios, the system forms a viscous suspension with poor fluidity due to insufficient solvent, which limits the contact area between enzymes and *perilla* seeds. This restricts enzymatic hydrolysis and results in lower oil yields [35]. When the ratio reached the optimal value of 9:1, the driving force for mass transfer between the solid and liquid phases was enhanced, thereby promoting oil release and increasing yield. However, beyond this optimal ratio, excessive dilution of both enzyme and substrate occurred, reducing effective enzyme–substrate collisions, lowering reaction rates, and complicating downstream oil separation [36,37].

Figure 3d illustrates the effect of enzyme concentration on oil yield, with an optimal concentration of 5%. Below this level, oil yield increased with increasing enzyme concentration due to improved enzyme–substrate interactions, which accelerated enzymatic hydrolysis and disrupted seed cell wall structures, thereby facilitating oil release [38]. Above 5%, oil yield decreased, likely due to excessive hydrolysis promoting interactions among polysaccharides, hydrolysis products (e.g., small-molecule sugars), and enzyme proteins. These interactions may alter enzyme conformation and surface properties, significantly enhancing their emulsifying activity [39]. In AEE systems, increased emulsification reduces interfacial tension between oil and water phases and stabilizes the interfacial film, leading to the formation of stable emulsions that hinder oil separation and ultimately reduce extraction efficiency [40].

### 2.3. Analysis and Validation of Response Surface Experiment Results

#### 2.3.1. Response Surface Analysis

Based on the results of the single-factor experiments, a response surface methodology (RSM) model was developed to optimize the extraction conditions of *perilla* seed oil. The independent variables included enzyme concentration (A, %), enzymatic hydrolysis temperature (B, °C), enzymatic hydrolysis time (C, h), and liquid-to-material ratio (D, mL·g^−1^), with oil yield (Y, %) serving as the response variable. Table 1 summarizes the levels of each factor used in the single-factor experiments. Table 2 presents the experimental design matrix and corresponding results for the RSM optimization. Table 3 provides the analysis of variance (ANOVA) for the regression model.

Multiple regression analysis of the experimental data yielded the following second-order polynomial equation (Equation (1)), which describes the relationship between the response variable and the independent variables:

Oil yield (%) = 33.84 + 0.5783A + 0.8342B + 0.4008C + 0.8633D + 0.5550AB + 0.2425AC + 0.1675AD + 0.6150BC − 0.2725BD + 0.2550CD − 2.08A^2^ − 1.31B^2^ − 2.09C^2^ − 1.32D^2^ (1)

As shown in Table 3, the regression model was highly significant (*p* < 0.0001), while the lack-of-fit test was not significant (*p* > 0.05), indicating that the model is statistically valid and well-fitted to the experimental data. The coefficient of determination (R^2^) was 0.9487, suggesting that approximately 94.87% of the variability in oil yield can be explained by the model. The adjusted R^2^ (R^2^_adj) was 0.8975, and the predicted R^2^ (R^2^_pred) was 0.7369, with a difference of less than 0.2, indicating good predictive reliability. In addition, the coefficient of variation (C.V.) was 1.79%, which is well below the acceptable threshold of 5%, further confirming the model’s precision and reliability [41]. Collectively, these statistical indicators demonstrate that the model is suitable for predicting *perilla* seed oil extraction yield.

**Figure 4:**
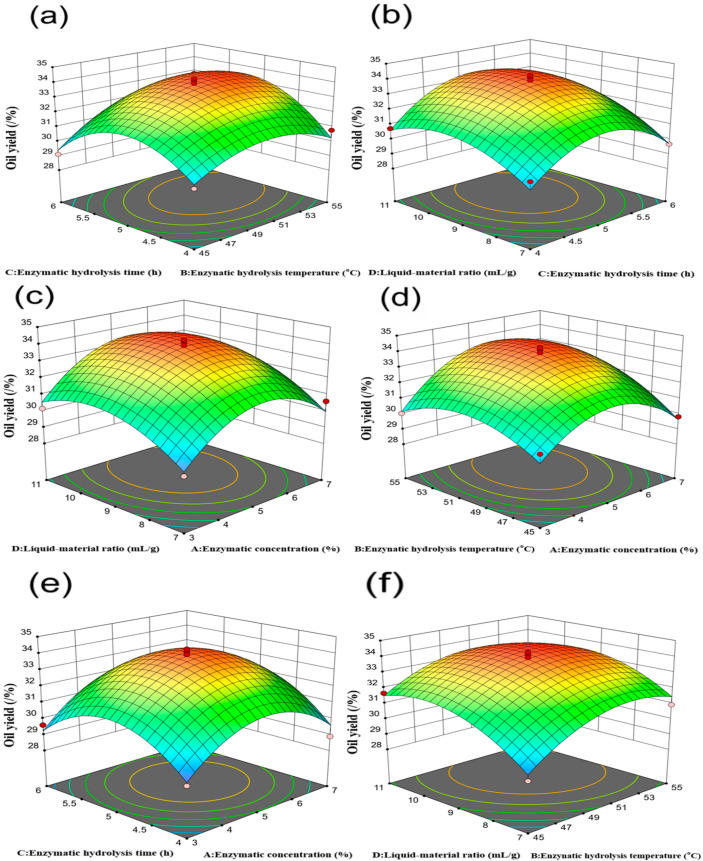
Response surface plots showing the effects of factor interactions on the oil yield of *perilla* seeds. (**a**) The interaction between enzymatic hydrolysis temperature and enzymatic hydrolysis time; (**b**) The interaction between enzymatic hydrolysis time and liquid-to-material ratio; (**c**) The interaction between enzyme concentration and liquid-to-material ratio; (**d**) The interaction between enzyme concentration and enzymatic hydrolysis temperature; (**e**) The interaction between enzyme concentration and enzymatic hydrolysis time; (**f**) The interaction between enzymatic hydrolysis temperature and liquid-to-material ratio.

Statistical analysis revealed that the linear terms (A, B, C, D), the interaction term BC, and the quadratic terms (A^2^, B^2^, C^2^, D^2^) were either significant (*p* < 0.05) or highly significant (*p* < 0.01), indicating that enzyme concentration, enzymatic hydrolysis temperature, enzymatic hydrolysis time, and liquid-to-material ratio all exert significant effects on oil yield. Figure 4 presents the response surface plots generated using Design-Expert 13.0 software. The interaction effects between pairs of independent variables can be interpreted based on the shape and steepness of the response surfaces and their corresponding contour plots [42]. Circular contours with relatively flat response surfaces indicate weak interactions and limited influence on the response, whereas elliptical contours with steep surfaces indicate strong interactions and more pronounced effects. Based on the F-values presented in Table 3, the significance ranking of pairwise interactions is as follows: BC (enzymatic hydrolysis temperature × enzymatic hydrolysis time) > AB (enzyme concentration × enzymatic hydrolysis temperature) > BD (enzymatic hydrolysis temperature × liquid-to-material ratio) > CD (enzymatic hydrolysis time × liquid-to-material ratio) > AC (enzyme concentration × enzymatic hydrolysis time) > AD (enzyme concentration × liquid-to-material ratio).

#### 2.3.2. Verification of the Optimal Response Surface

The model-predicted optimal conditions for UEAEE of *perilla* seed oil were an enzyme concentration of 5.425%, an enzymatic hydrolysis temperature of 51.87 °C, an enzymatic hydrolysis time of 5.18 h, and a liquid-to-material ratio of 9.636:1 (mL/g). Under these conditions, the predicted oil yield was 34.23%. To facilitate practical operation and account for equipment precision, the optimized parameters were adjusted to an enzyme concentration of 5%, a temperature of 52 °C, a hydrolysis time of 5 h, and a liquid-to-material ratio of 9.5:1 (mL/g). Validation experiments conducted in five parallel runs under these adjusted conditions yielded an average oil extraction rate of 34.20%, as shown in Table 4. The minimal deviation between the experimental and predicted values indicates a high degree of agreement, demonstrating that the response surface model provides an accurate representation of the experimental system. These results confirm that the optimized process parameters are stable and highly reproducible, offering reliable guidance for practical and industrial applications.

### 2.4. Effect of Different Extraction Methods on Oil Yield of Perilla Seeds

As shown in Table 5, response surface optimization increased the oil yield of *perilla* seeds to 34.20% using the UEAEE method. This yield is comparable to that obtained by (SE, 34.81%) and significantly higher than that achieved by (PE, 28.77%). These results confirm the effectiveness of ultrasound-assisted technology in enhancing mass transfer and disrupting cellular structures. Compared with the ultrasound-assisted AEE method reported by Li et al. [11], which achieved an oil yield of 31.34%, the incorporation of ethanol in the pretreatment stage further improves extraction efficiency. The core advantage of the UEAEE method lies in the synergistic effects of ethanol-mediated chemical solubilization, enzyme-specific hydrolysis, and ultrasound-induced cavitation. Unlike cold pressing, which involves limited cell disruption and incomplete oil release, UEAEE achieves a significantly higher extraction efficiency. Compared with SE, which requires continuous heating and may lead to degradation of heat-sensitive bioactive compounds, UEAEE operates under milder conditions, better preserves functional constituents, and demonstrates superior overall performance.

### 2.5. Effect of Different Extraction Methods on the Quality of Perilla Seed Oil

#### 2.5.1. Fatty Acid Composition Analysis

Table 6 presents the fatty acid composition of *perilla* seed oil extracted using different methods. The primary fatty acids identified were ALA, linoleic acid, oleic acid, palmitic acid, and stearic acid. ALA was the predominant fatty acid, ranging from 71.09% to 71.82%, followed by linoleic acid (12.19–12.46%), oleic acid (10.07–10.28%), palmitic acid (4.90–5.30%), and stearic acid (0.67–0.95%). This composition and relative abundance are consistent with the findings reported by Zamani et al. [43]. Minor variations in individual fatty acid contents may be attributed to differences in seed origin, growing conditions, harvest batches, and pretreatment methods.

Further analysis revealed that the content of unsaturated fatty acids was significantly higher than that of saturated fatty acids in all samples. The highest proportion of unsaturated fatty acids, reaching 94.43%, was observed in oil extracted by UEAEE, highlighting its advantage in preserving beneficial lipid components. The ALA content showed only slight variation among the three extraction methods: 71.70% for UEAEE, 71.82% for PE, and 71.09% for SE. This trend is consistent with the findings of Hao et al. [3], who reported higher ALA retention in oil obtained by PE compared with SE. Similarly, Li et al. [44] demonstrated that ultrasound-assisted extraction yields higher ALA levels than SE. Overall, while ALA content remained relatively stable across the extraction methods, UEAEE exhibited a higher total unsaturated fatty acid content compared with both SE and PE. This suggests that UEAEE is more effective in preserving the nutritional quality and overall fatty acid profile of *perilla* seed oil.

#### 2.5.2. Quality Analysis

AV and peroxide value (POV) are key indicators used to evaluate oil quality. AV reflects the extent of hydrolytic rancidity, with higher values indicating greater levels of free fatty acids, while POV measures primary oxidative rancidity, where elevated values correspond to increased lipid oxidation. In general, higher values of both parameters indicate deteriorated oil quality. As shown in Table 7, the AV of oil extracted by UEAEE (0.89 mg/g) was significantly lower than that obtained by (SE, 1.42 mg/g), indicating that UEAEE effectively suppresses the formation of free fatty acids. The higher AV observed in SE oil may be attributed to the combined effects of prolonged heating and organic solvent exposure, which can enhance endogenous lipase activity, accelerate triglyceride hydrolysis, and consequently increase AV [45]. Similarly, the POV of UEAEE oil (0.01 g/100 g) was markedly lower than that of SE oil (0.21 g/100 g). The elevated POV in SE oil may result from prolonged thermal exposure, solvent effects, and increased oxygen availability during extraction, all of which promote lipid oxidation. In contrast, the milder processing conditions and reduced oxygen exposure associated with UEAEE effectively inhibit the formation of primary oxidation products, thereby better preserving oil quality.

The carotenoid content, a key group of natural lipophilic antioxidants, varied depending on the extraction method. This study compared carotenoid levels in oils obtained via UEAEE, PE, and SE. The highest carotenoid content was observed in UEAEE-extracted oil (1.23 mg/kg), followed by PE (1.11 mg/kg), with SE yielding the lowest value (0.93 mg/kg). Although these differences were not statistically significant, the improved carotenoid retention in UEAEE is likely attributable to ultrasonic cavitation effects. In combination with enzymatic hydrolysis, ultrasound enhances cell wall disruption, thereby facilitating the more efficient release of intracellular carotenoids [25]. This mechanism may explain the higher carotenoid content observed in UEAEE-extracted oil.

The total flavonoid content (TFC) reached its highest value in UEAEE-extracted oil (1.04 mg/g), which was approximately 3.15 times higher than that obtained by SE (0.33 mg/g) and significantly higher than that obtained by PE (0.36 mg/g). These results indicate that UEAEE is more effective in promoting the dissolution and release of flavonoid compounds. According to Jia [46], heat pretreatment reduces seed moisture content, increases brittleness, and enhances cell wall disruption, thereby improving permeability and facilitating the release of bound flavonoids. Furthermore, ultrasound intensifies heat and mass transfer, further promoting the release of intracellular compounds. The synergistic effects of these processes contribute to the significantly higher flavonoid content observed in UEAEE-extracted oil.

As shown in Table 7, the total phenolic content (TPC) of *perilla* seed oil followed the order UEAEE (2.79 mg/g) > SE (1.88 mg/g) > PE (1.77 mg/g). This variation is likely associated with differences in the efficiency of phenolic compound release under different extraction conditions. In the UEAEE process, the enhanced extraction of phenolic compounds is primarily attributed to ultrasonic cavitation. The collapse of cavitation bubbles generates intense shock waves and shear forces, which disrupt plant cell wall structures and facilitate the release of phenolic compounds [25,47]. Similarly, Esmaeilzadeh et al. [48] reported that ultrasound treatment effectively promotes the release of phenolic compounds from hemp seed oil, further confirming the advantage of the UEAEE method in enhancing TPC.

The DPPH free radical scavenging activity is a widely used indicator of antioxidant capacity in plant extracts and bioactive compounds. As shown in Table 7, the DPPH scavenging rates differed significantly among the oils extracted by different methods: UEAEE (80.60%) > SE (73.20%) > PE (70.15%). The superior antioxidant activity of UEAEE-extracted oil can be attributed to ultrasonic cavitation, where the collapse of cavitation bubbles generates localized high temperature, high pressure, and strong shear forces that effectively disrupt plant cell structures and promote the release of intracellular bioactive compounds [49]. Consequently, UEAEE oil contains higher levels of antioxidant constituents, such as phenolic compounds and carotenoids, which contribute to its enhanced free radical scavenging capacity. Notably, all *perilla* seed oils exhibited DPPH scavenging activities above 70%, indicating strong antioxidant potential. These findings provide a solid experimental basis for the further development and application of *perilla* seed oil as a natural antioxidant source.

## 3. Materials and Methods

### 3.1. Materials and Reagents

*Perilla* seeds were sourced from Heihe City, Heilongjiang Province, China (47.70–51.05° N, 124.75–129.30° E). Mature seeds with full kernels, uniform size, and no signs of decay, mold, or shell damage were selected for use. The moisture content (7.67 ± 0.05%) and oil content (38.91 ± 0.12%) of the raw materials were determined according to GB/T 14489.1-2008 Oilseeds, Determination of Moisture and Volatile Matter Content [50] and GB/T 14488.1-2008 Oilseeds, Determination of Oil Content [51], respectively.

All reagents used in this study were of analytical grade. Alcalase (enzyme activity: 200,000 U/g) was purchased from Fuzhou Phygene Biotechnology Co., Ltd. (Fuzhou, China). Ethanol, n-hexane, methanol, gallic acid, anhydrous sodium sulfate, sodium chloride, and hydrochloric acid were obtained from Tianjin Zhiyuan Reagent Co., Ltd. (Tianjin, China). Isooctane, sodium hydrogen sulfate, and potassium hydroxide were supplied by Tianjin Fuchen Chemical Reagent Factory (Tianjin, China). Petroleum ether was provided by Fuchen (Tianjin) Chemical Reagent Co., Ltd. (Tianjin, China), and diethyl ether was purchased from Hunan Huihong Reagent Co., Ltd. (Changsha, China). Sodium hydroxide was obtained from Tianjin Hengxing Chemical Reagent Manufacturing Co., Ltd. (Tianjin, China). 1,1-diphenyl-2-picrylhydrazyl (DPPH) and Folin–Ciocalteu reagent were purchased from Fuzhou Phygene Biotechnology Co., Ltd. (Fuzhou, China), while rutin was obtained from Shanghai Qiangshun Chemical Reagent Manufacturing Co., Ltd. (Shanghai, China).

### 3.2. Experimental Methods

Sample Preparation: Mature, healthy *perilla* seeds with full kernels, uniform size, and no visible signs of decay, mold, or shell damage were selected. The seeds were evenly spread on a stainless steel tray and dried in a constant-temperature oven at 40 °C until a constant weight was achieved. After cooling to room temperature, the dried seeds were ground into powder and passed through a 60-mesh sieve. The resulting fine powder was sealed in sample bags and stored at −4 °C until further analysis.

#### 3.2.1. Ultrasound–Ethanol Pretreatment

A 10 g portion of sample powder was mixed with 30 mL of 60% (*v*/*v*) ethanol solution [15], ensuring complete immersion of the material. The mixture was then subjected to ultrasonic pretreatment at 55 °C, 40 kHz, and 200 W for 10, 20, 30, 40, or 50 min. After pretreatment, the mixture was filtered, and the residue was collected and dried for subsequent use.

*Perilla* seed oil samples obtained under different pretreatment conditions were divided into four experimental groups. Group I consisted of oil extracted without ethanol addition and without ultrasonic pretreatment. Group II included oil extracted with ethanol addition but without ultrasonic pretreatment. Group III included oil extracted with ultrasonic pretreatment but without ethanol addition. Group IV consisted of oil extracted with both ethanol addition and ultrasonic pretreatment.

#### 3.2.2. Scanning Electron Microscopy (SEM)

The microstructural changes in *perilla* seeds subjected to different pretreatment conditions were examined using SEM (Carl Zeiss AG ZEISS EVO 15, Oberkochen, Germany). Prior to imaging, the samples were vacuum-dried and sputter-coated with a thin layer of gold-palladium alloy to enhance electrical conductivity. SEM observations were performed at an accelerating voltage of 20.0 kV and a magnification of 2000×.

#### 3.2.3. Ultrasound–Ethanol Pretreatment Combined with AEE (UEAEE)

UEAEE was performed by combining ultrasound–ethanol pretreatment with AEE. At a predetermined liquid-to-material ratio, the pretreated *perilla* seed sample was mixed with alcalase at a specified concentration. Based on preliminary experiments and the findings of Yuan Decheng et al. [52], alcalase was selected for the enzymatic hydrolysis of *perilla* seeds. The pH was adjusted to 9.5 using 0.1 mol/L HCl or 0.1 mol/L NaOH, as alcalase exhibits optimal catalytic activity within the pH range of 9.0–11.5. This pH condition ensures structural stability of the enzyme active site and enhances substrate binding; the specific value of 9.5 was selected based on preliminary optimization (Figure 1c). Enzymatic hydrolysis was then carried out in a constant-temperature shaker at the designated temperature for a predetermined duration. After hydrolysis, the enzyme was inactivated by heating the mixture in a boiling water bath at 100 °C for 10 min. The mixture was then centrifuged at 6000× *g* for 20 min, and the upper oil phase was collected (M_1_). The emulsion phase was subsequently frozen at −18 °C for 24 h, thawed at room temperature for 2 h, and centrifuged again at 6000× *g* for 10 min to obtain additional oil (M_2_). Finally, M1 and M2 were combined and stored at 4 °C for further analysis. The oil yield was calculated using Equation (2):

(2) $$\mathrm{Oil}\;\mathrm{yield}\;(\%)=\frac{M_{1}+M_{2}}{M_{0}}\times 100\%$$

where M_0_ is the mass of the raw material (g), M_1_ is the mass of oil recovered from the first centrifugation (g), and M_2_ is the mass of oil recovered from the second centrifugation (g).

#### 3.2.4. Single-Factor Experiments

Single-factor experiments were conducted to investigate the effects of four variables on the oil yield of *perilla* seeds during the enzymatic hydrolysis stage of the UEAEE process. The variables examined included enzymatic hydrolysis time, temperature, enzyme concentration, and liquid-to-material ratio. Hydrolysis time was varied at 3, 4, 5, 6, and 7 h; temperature was set at 40, 45, 50, 55, and 60 °C; enzyme concentration was adjusted to 1%, 3%, 5%, 7%, and 9%; and liquid-to-material ratios of 3:1, 5:1, 7:1, 9:1, and 11:1 (mL/g) were evaluated. All experiments were performed in triplicate, and the results are expressed as mean values.

#### 3.2.5. Experimental Design of Response Surface Optimization

Building on the results of the single-factor experiments, a Box–Behnken design was employed to perform RSM optimization. The independent variables included enzyme concentration, enzymatic hydrolysis time, temperature, and liquid-to-material ratio, while the response variable was the oil yield of *perilla* seeds.

#### 3.2.6. Extraction by PE

Referring to the method reported by Zeng et al. [53], *perilla* seeds were subjected to screw pressing at 50 ± 5 °C under a pressure of 50 MPa. The crude oil obtained was then centrifuged at 6000 rpm for 10 min to remove residual solids. The clarified oil was sealed and stored at −4 °C until further analysis.

#### 3.2.7. Extraction by SE

Referring to the method reported by Zeng et al. [53], *perilla* seed powder was wrapped in filter paper and placed in a Soxhlet extractor. Petroleum ether was used as the extraction solvent, and extraction was carried out at 90 °C for 6 h. The resulting extract was collected in a round-bottom flask, and the solvent was removed using a rotary evaporator. The obtained oil was then dried in an oven at 100 °C to constant weight and stored at −4 °C until further analysis.

### 3.3. Quality Analysis of Perilla Seed Oil

#### 3.3.1. Fatty Acid Composition

The fatty acid composition was determined according to GB 5009.168-2016 National Food Safety Standard, Determination of Fatty Acids in Foods [54].

#### 3.3.2. AV and POV

The AV of *perilla* seed oil was determined according to GB 5009.229-2016 National Food Safety Standard, Determination of AV in Foods [55]. The POV was measured following GB 5009.227-2023 National Food Safety Standard, Determination of POV in Foods [56].

#### 3.3.3. Carotenoid Content

Carotenoid content was measured according to the method described by [57]. Briefly, 0.2 g of oil sample was accurately weighed, dissolved, and diluted to a fixed volume with petroleum ether. The absorbance of the resulting solution was then measured at 450 nm. Carotenoid content was calculated using the following formula Equation (3):

(3) $$\mathrm{Carotenoid}\;\mathrm{content}\;(\mathrm{mg}/\mathrm{kg})=\frac{1000\;\times \;10\;\times \;E}{A\;\times \;m\;(g)}$$

where *E* is the absorbance; *A* is the average extinction coefficient of carotenoids, 2500; m is the sample mass (g).

#### 3.3.4. TPC

TPC was determined according to the method described by [58]. Briefly, 2.5 g of oil sample was mixed with 2.5 mL of 80% ethanol in a conical flask, vortexed for 1 min, and subjected to ultrasonic extraction at 50 °C for 30 min. After cooling under running water, the mixture was centrifuged at 10,000 rpm for 10 min, and the supernatant was collected for analysis. Then, 1 mL of the supernatant was transferred into a colorimetric tube and thoroughly mixed with 3.5 mL of 0.1 mol/L Folin–Ciocalteu reagent. After standing for 3 min, 2.5 mL of 7.5% sodium carbonate solution was added, and the volume was adjusted to 10 mL with distilled water. The mixture was incubated in the dark for 1 h, after which the absorbance was measured at 765 nm.

A gallic acid stock solution (1.0 mg/mL) was prepared and diluted to obtain standard solutions with concentrations of 5, 10, 20, 30, 40, and 50 μg/mL. The absorbance of these standards was measured using the same procedure. A calibration curve was constructed by plotting gallic acid concentration (X) against absorbance (Y), yielding the linear regression equation: Y = 0.0013X + 0.1061 (R^2^ = 0.9928). The TPC of samples was calculated by substituting the absorbance values into this equation.

#### 3.3.5. TFC

TFC was determined according to the method described by [59]. Rutin (40 mg) was dissolved in 60% ethanol and diluted to 100 mL to obtain a stock solution with a concentration of 400 mg/L. Aliquots of 0, 1, 2, 3, and 4 mL of the stock solution were transferred into separate 25 mL volumetric flasks, and each flask was supplemented with 4 mL of 60% ethanol. Subsequently, 1 mL of 5% NaNO_2_ solution, 1 mL of 10% Al(NO_3_)_3_ solution, and 10 mL of 4% NaOH solution were added sequentially, with thorough mixing and a 6 min standing period after each addition. The volume was then adjusted to the mark with 60% ethanol, and the mixtures were allowed to stand for an additional 15 min. Absorbance was measured at 510 nm. A standard curve was constructed by plotting rutin concentration (X) against absorbance (Y), yielding the linear regression equation: Y = 0.4946X + 0.0201 (R^2^ = 0.9913). The TFC of the samples was calculated by substituting absorbance values into this equation. Results are expressed as rutin equivalents.

#### 3.3.6. DPPH Free Radical Scavenging Rate

The DPPH free radical scavenging activity was determined according to the method described by [60]. Briefly, 0.5 g of the oil sample was dissolved and diluted with absolute ethanol to 5 mg/mL for analysis. A 0.1 mmol/L DPPH solution was prepared using absolute ethanol as the solvent. The 2 mL of oil sample solution was then mixed with the 2 mL of DPPH solution and incubated in the dark for 30 min. Subsequently, the absorbance was measured at 517 nm. The DPPH radical scavenging rate was calculated using the following formula Equation (4):

(4) $$\mathrm{Radical}\;\mathrm{Scavenging}\;\mathrm{Rate}\;(\%)=\frac{A_{3}-\left(A_{1}-\left.A_{2}\right)\right.}{A_{3}}\times 100\%$$

where *A*_1_ is the absorbance of the mixture containing 2 mL of sample solution and 2 mL of DPPH solution; *A*_2_ is the absorbance of the mixture containing 2 mL of sample solution and 2 mL of absolute ethanol; and *A*_3_ is the absorbance of the mixture containing 2 mL of absolute ethanol and 2 mL of DPPH solution.

### 3.4. Data Analysis

All experiments were conducted in triplicate, and results are reported as arithmetic means. The effects of independent variables on oil yield were systematically analyzed using Origin 2021 and Design-Expert 13 software. The physicochemical properties of the extracted oils were characterized, and one-way ANOVA was performed using SPSS 27 software for statistical evaluation. Data are presented as mean ± standard deviation, and statistical significance was defined at *p* < 0.05.

## 4. Conclusions

UEAEE was applied to extract oil from *perilla* seeds, and the results demonstrate that ultrasound–ethanol pretreatment significantly enhances oil yield by improving solvent penetration and facilitating oil release compared with untreated samples. Moreover, *perilla* seed oil extracted via UEAEE exhibited superior quality, characterized by a high unsaturated fatty acid content (94.43%), low acid and POVs, elevated levels of total flavonoids and total phenols, and strong DPPH free radical scavenging activity. In summary, UEAEE offers dual advantages by improving extraction efficiency and enhancing oil quality, providing strong technical support for the green and efficient extraction and potential industrial application of *perilla* seed oil. However, current limitations of this technology remain, including limited process adaptability, insufficient oxidative stability of the oil, challenges in scale-up, and high energy consumption associated with ethanol recovery. Future research should focus on precise regulation of process parameters, integration of artificial intelligence for process modeling, and development of dedicated equipment systems. These efforts will improve oil stability and extraction efficiency and promote the industrial application of this technology.

## Figures and Tables

**Table 1:** Single-factor level.

Level	A (%)	B (°C)	C (h)	D (mL/g)
−1	3	45	4	7:1
0	5	50	5	9:1
1	7	55	6	11:1

**Table 2:** Optimization Design and Results of the response Surface.

Experimental Number	A (%)	B (°C)	C (h)	D (mL/g)	Oil Yield/%
1	3	45	5	9:1	30.16 ± 0.15
2	7	45	5	9:1	29.83 ± 0.13
3	3	55	5	9:1	30.02 ± 0.18
4	7	55	5	9:1	31.94 ± 0.12
5	5	50	4	7:1	29.99 ± 0.14
6	5	50	6	7:1	29.83 ± 0.15
7	5	50	4	11:1	30.71 ± 0.13
8	5	50	6	11:1	31.59 ± 0.16
9	3	50	5	7:1	28.90 ± 0.12
10	7	50	5	7:1	30.57 ± 0.18
11	3	50	5	11:1	30.15 ± 0.14
12	7	50	5	11:1	32.53 ± 0.16
13	5	45	4	9:1	29.63 ± 0.23
14	5	55	4	9:1	30.78 ± 0.15
15	5	45	6	9:1	29.15 ± 0.17
16	5	55	6	9:1	32.80 ± 0.13
17	3	50	4	9:1	28.77 ± 0.19
18	7	50	4	9:1	28.86 ± 0.14
19	3	50	6	9:1	29.62 ± 0.16
20	7	50	6	9:1	30.72 ± 0.11
21	5	45	5	7:1	28.86 ± 0.12
22	5	55	5	7:1	30.95 ± 0.17
23	5	45	5	11:1	31.67 ± 0.15
24	5	55	5	11:1	32.68 ± 0.20
25	5	50	5	9:1	33.59 ± 0.12
26	5	50	5	9:1	34.13 ± 0.10
27	5	50	5	9:1	33.30 ± 0.13
28	5	50	5	9:1	33.95 ± 0.16
29	5	50	5	9:1	34.28 ± 0.19

**Table 3:** ANOVA of the regression model.

Project	Sum of Squares	Freedom	Mean Square	F-Value	*p*-Value	Significance
Model	79.54	14	5.68	18.51	<0.0001	**
A—Enzyme concentration	4.01	1	4.01	13.08	0.0028	*
B—Enzymatic hydrolysis temperature	8.35	1	8.35	27.21	0.0001	**
C—Enzymatic hydrolysis time	1.93	1	1.93	6.28	0.0252	*
D—Liquid-to-material ratio	8.94	1	8.94	29.14	0.0001	**
AB	1.23	1	1.23	4.01	0.0649	
AC	0.2352	1	0.2352	0.7664	0.3961	
AD	0.1122	1	0.1122	0.3656	0.5551	
BC	1.51	1	1.51	4.93	0.0434	*
BD	0.2970	1	0.2970	0.9678	0.3419	
CD	0.2601	1	0.2601	0.8475	0.3729	
A^2^	28.14	1	28.14	91.68	<0.0001	**
B^2^	11.07	1	11.07	36.07	0.0001	**
C^2^	28.37	1	28.37	92.45	<0.0001	**
D^2^	11.35	1	11.35	36.98	0.0001	**
Lack-of-fit error	3.66	10	0.3656	2.28	0.2215	
Pure error	0.6409	4	0.1602			
Sum	83.84	28				

Note: * indicates a significant difference (*p* < 0.05); ** indicates a highly significant difference (*p* < 0.01).

**Table 4:** Process verification of the optimal response surface.

Group	1	2	3	4	5	Average
Oil yield/%	34.19	33.98	34.12	34.38	34.31	34.20 ± 0.16

**Table 5:** Different Extraction Methods on the Oil Yield of *Perilla* Seeds.

	UEAEE	SE	PE
Oil yield (%)	34.20 ± 0.16 ^a^	34.81 ± 0.72 ^a^	28.77 ± 0.21 ^b^

Note: Different lowercase letters in the Table indicate statistically significant differences (*p* < 0.05). The same notations apply to the following all Table.

**Table 6:** Relative content of fatty acids by different extraction methods.

Fatty Acid	UEAEE/%	SE/%	PE/%
Palmitic acid (16:0)	4.90 ± 0.06 ^c^	5.30 ± 0.03 ^a^	5.11 ± 0.01 ^b^
stearic acid (18:0)	0.67 ± 0.16 ^c^	0.95 ± 0.05 ^a^	0.82 ± 0.07 ^b^
oleic acid (18:1)	10.28 ± 0.02 ^a^	10.23 ± 0.02 ^a^	10.07 ± 0.06 ^b^
Linoleic acid (18:2)	12.46 ± 0.02 ^a^	12.43 ± 0.29 ^a^	12.19 ± 0.04 ^b^
α-Linolenic acid (18:3w3)	71.70 ± 0.10 ^a^	71.09 ± 0.23 ^b^	71.82 ± 0.01 ^a^
Saturated fatty acid	5.57 ± 0.11 ^c^	6.25 ± 0.07 ^a^	5.93 ± 0.09 ^b^
Unsaturated fatty acid	94.43 ± 0.11 ^a^	93.75 ± 0.07 ^c^	94.07 ± 0.09 ^b^

**Table 7:** Quality analysis indicators of various extraction methods.

Indicator	UEAEE	SE	PE
Acid value (mg/g)	0.89 ± 0.05 ^c^	1.42 ± 0.03 ^a^	1.03 ± 0.03 ^b^
Peroxide value (g/100 g)	0.01 ± 0.01 ^c^	0.21 ± 0.01 ^a^	0.03 ± 0.03 ^b^
Carotenoid content (mg/kg)	1.23 ± 0.06 ^a^	0.93 ± 0.05 ^c^	1.11 ± 0.04 ^b^
Total flavonoids (mg/g)	1.04 ± 0.09 ^a^	0.33 ± 0.02 ^b^	0.36 ± 0.05 ^b^
Total phenolics (mg/g)	2.79 ± 0.07 ^a^	1.88 ± 0.02 ^b^	1.77 ± 0.07 ^b^
DPPH radical scavenging ability (%)	80.60 ± 0.71 ^a^	73.20 ± 2.26 ^b^	70.15 ± 3.32 ^b^

## Data Availability

All data generated or analyzed during this study are included in this published article.
